# The Primary Cilium as a Therapeutic Target in Ocular Diseases

**DOI:** 10.3389/fphar.2020.00977

**Published:** 2020-06-26

**Authors:** Peng Zhou, Jun Zhou

**Affiliations:** ^1^ Institute of Biomedical Sciences, Shandong Provincial Key Laboratory of Animal Resistance Biology, Collaborative Innovation Center of Cell Biology in Universities of Shandong, College of Life Sciences, Shandong Normal University, Jinan, China; ^2^ State Key Laboratory of Medicinal Chemical Biology, College of Life Sciences, Nankai University, Tianjin, China

**Keywords:** primary cilium, eye, cornea, retina, ocular disease, gene therapy, stem cell therapy

## Abstract

Primary cilia are microtubule-based cellular structures located on the surfaces of most mammalian cells and play important roles in detecting external stimuli, signal transduction, and cell cycle regulation. Primary cilia are also present in several structures of the eye, and their abnormal development or dysfunction can cause various ocular diseases. The rapid development of proteomics and metabolomics technologies have helped in the identification of many ocular disease-related proteins, some of which are dysregulated in primary cilia. This review focuses on ciliary dysregulation in a number of ocular diseases and discusses the potential of targeting primary cilia in gene and stem cell therapy for these diseases.

## Introduction

The eye converts light stimulation into nerve impulses that are transmitted to the brain *via* the optic nerve. The main outer wall of the eyeball has three membranous layers. The outer membrane layer is composed of fibrous connective tissue and the transparent cornea, the sclera, and the specialized limbus between the cornea and the sclera containing limbal stem cells. The middle membranous layer is rich in blood vessels and pigment cells divided from front to back into the iris, ciliary body, and choroid. The inner membrane layer or retina is the important photosensitive layer of the eye made up from photoreceptor cells in some but not all parts connected to the choroid. The eye contains aqueous humor, the lens, and vitreous, which form a refractive system together with the cornea. The eye is surrounded by accessory structures including the lacrimal gland, the lacrimal duct, and the eyelid, which protect the eyeball and ensure visual stability (DelMonte and Kim, 2011).

**Figure 1:**
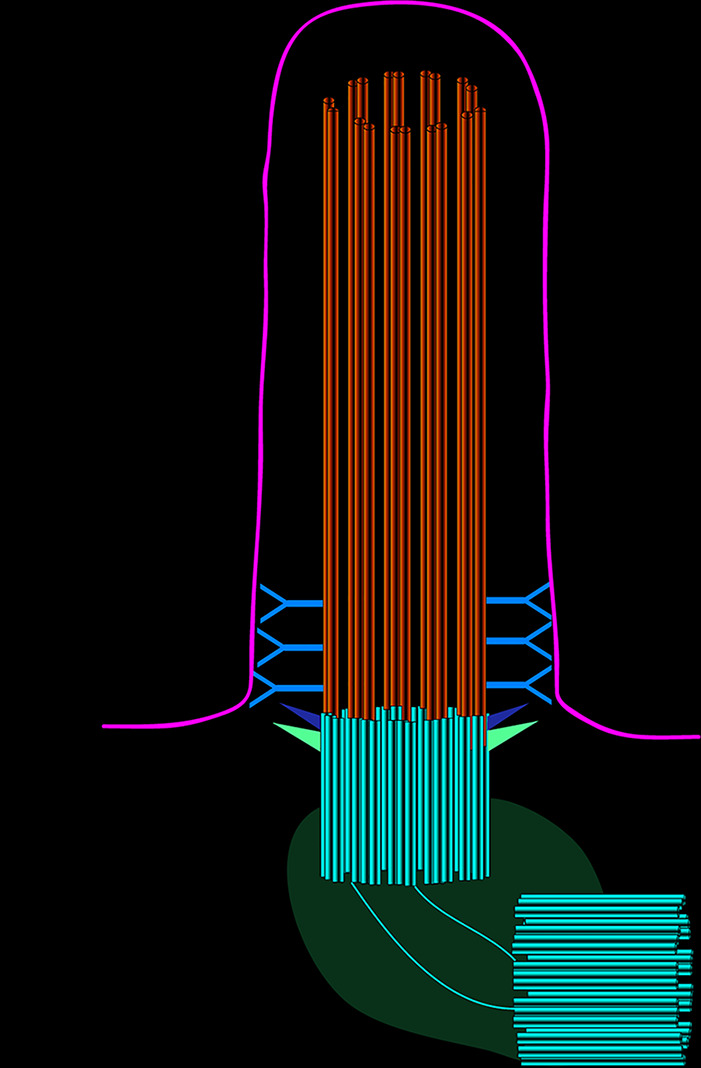
Structural organization of the primary cilium. The primary cilium is assembled from a basal body, an axoneme, and a ciliary membrane. Between the basal body and axoneme is the transitional zone.

All three layers of the eyeball contain primary cilia, which play important roles in maintaining normal eye function (May-Simera et al., 2017). Almost all cells have primary cilia, which consist of an axoneme, a basal body, the transition zone, and the ciliary membrane (**Figure 1**). Unlike motile cilia, primary cilia only have nine doublet microtubules at the periphery of the axonemes and do not have two central microtubules. Therefore, primary cilia do not have a motility function but instead have a sensory function through various signaling pathways (Bangs and Anderson, 2017; Lyu and Zhou, 2017; Yang et al., 2019). Loss of primary cilia is associated with several pathologies, and their loss is also implicated in retinopathy. While most of the current evidence on primary cilia in ocular diseases focuses on retinal photoreceptor cells, primary cilia have also been detected in the cornea, limbus, ciliary body, lens, and retinal pigment epithelial (RPE) cell layer (Blitzer et al., 2011; Grisanti et al., 2016; Yu et al., 2019). With recent rapid developments in high-throughput technologies, an increasing number of cilium-related proteins have been identified, laying the foundation for exploring the relationships between primary cilia and ocular diseases and providing new opportunities for the genetic diagnosis and treatment of ophthalmic diseases. In this review, we describe the cilium-related molecules implicated in ocular diseases and how they can be exploited for therapeutic benefit.

## Primary Cilia of Ocular Tissues

**Figure 2:**
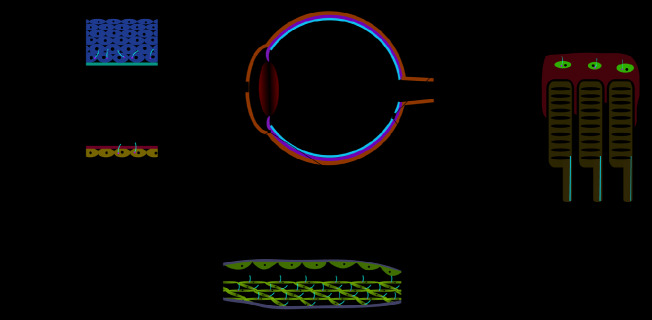
Localization of primary cilia in mammalian eyes. **(A)** The multi-layered structure of the cornea. Primary cilia are located on the basal cells of the corneal epithelium and corneal endothelial cells and, as endothelial cells mature, the primary cilia gradually reduce in number until they disappear. **(B)** The structure of the trabecular meshwork, located at the limbus of the anterior chamber angle, and the primary cilia on the trabecular meshwork cells can respond to the pressure of the aqueous humor. **(C)** Structure of the lateral part of the retina. Primary cilia are present on the retinal pigment epithelium and disappear after the epithelium matures. The outer segment of the photoreceptor is connected to the inner segment by a connecting cilium.

Primary cilia are present in both the epithelial and endothelial cell layers of the cornea and play a specific role in corneal development (**Figure 2A**). The anterior chamber contains a specialized structure known as the trabecular meshwork, which controls the flow of aqueous humor and on which primary cilia are found (Sacca et al., 2016). Primary cilia on trabecular meshwork cells can sense aqueous humor flow and therefore play an important role in regulating intraocular pressure, and excessive intraocular pressure caused by obstruction of aqueous humor outflow underlies glaucoma (**Figure 2B**) (Sundaresan et al., 2019). When the intraocular pressure is too high, primary cilia shorten, promoting the expression of tumor necrosis factor α (TNF-α), and transforming growth factor β (TGF-β), thereby initiating the molecular mechanisms underpinning glaucoma (Luo et al., 2014).

The inner membrane layer comprises the RPE and the neurosensory layer, which can be subdivided into photoreceptors, bipolar cells, and ganglion cells. There are numerous reports about primary cilia on RPE and photoreceptors (**Figure 2C**). The outer segments of photoreceptor cells are usually regarded as specialized primary cilia, which are connected to the inner side through the transition zone (so-called connecting cilia) (Bachmann-Gagescu and Neuhauss, 2019). The RPE is located on the outermost side of the retina, and its maturity determines whether the outer segment of the photoreceptor is fully developed. Abnormal development of the outer segment is a major cause of retinal inflammation; inhibiting primary ciliary function results in a failure of maturation of induced pluripotent stem cell (iPSC)-RPE cells, and primary cilia control RPE cell maturation and retinal inflammation by regulating Wnt signaling. Since the visual system relies on converting light signals into electrophysiological stimuli, it is essential that primary cilia develop normally on photoreceptors (May-Simera et al., 2018). Changes in primary cilium morphology or structure can cause macular degeneration, diabetic retinopathy, and other ocular diseases, and recent studies have shown that abnormal cilia can also induce retinal neovascularization.

### Keratitis

Keratitis is corneal inflammation caused by infections such as bacteria and viruses. In the keratitis inflammatory response, the mitogen-activated protein kinase (MAPK) cascade and nuclear factor κB (NF-κB) pathways are activated, resulting in the production of the proinflammatory cytokines, which are mostly negatively correlated with primary cilia (Zhu et al., 2017). In the context of lung epithelial cells, interleukin 6 (IL-6) can stimulate the inhibitor of signal transducer and activator of transcription 3 (STAT3), and decreases in STAT3 levels leads to cilium loss on basal cells (Tadokoro et al., 2014). Lipopolysaccharide (LPS), an important bacterial membrane component, can increase the activity of histone deacetylases (HDACs), and HDAC6 expression induced by TGF-β deforms and shortens primary cilia and reduces cilium numbers (Ran et al., 2015; Ehnert et al., 2017; Zhang et al., 2019). HDAC6 is also a downstream substrate of Aurora A (Yu et al., 2016). In addition, the cylindromatosis deubiquitinase and apoptosis signal regulating kinase 1 promote cilium growth through HDAC6 (Yang et al., 2014; Ran et al., 2016; Ran et al., 2020). Mutations in X-pro-aminopeptidase 3 have also been detected in families with nephronophthisis (NPHP), an autosomal recessive disorder and classic ciliary disease that results in nephritis. Ten pathogenic genes (NPHP1-NPHP9 and NPHP11) are located on primary cilia, most of which are located in the transition zone where they play a gating role (O’Toole et al., 2010).

### Glaucoma

Glaucoma is a type of optic neuropathy characterized by elevated intraocular pressure (Shah, 2019). Therefore, the adjustment of intraocular pressure is particularly important for the treatment of glaucoma. As noted above, the primary cilia of the trabecular meshwork shorten in response to fluid flow and elevated aqueous humor pressure (Singh et al., 2019). Lowe syndrome is an X-linked congenital disease that has glaucoma as one of its typical manifestations. Patients with Lowe syndrome harbor mutations in oculo-cerebro-renal syndrome of Lowe (OCRL), and OCRL is involved in vesicle transport in primary cilia (Zheng et al., 2019). OCRL and transient receptor potential cation channel subfamily V member 4 (TRPV4) knockout mice also exhibit increased intraocular pressure and ciliary shortening (Luo et al., 2014). Bardet-Biedl****syndrome 5 (BBS5) has also been found to be localized in trabecular meshwork cilia. Microarray analysis and dual luciferase assays confirmed that BBS5 is regulated by the transcription factor paired like homeodomain 2 (PITX2), and the absence of PITX2 not only activates pro-inflammatory cytokines, but also reduces BBS5 to damage cilia (Moazzeni et al., 2016).

High-density protein microarrays performed on the sera of patients with secondary open-angle glaucoma showed that some proteins, such as EH domain containing 1 (EHD1) and fibroblast growth factor receptor 3 (FGFR3), were involved in early cilium formation (McNally and O’Brien, 2014). In the early stage of cilium formation, cilium-related proteins are transported from the Golgi apparatus to the top of the central body in vesicles to form ciliary vesicles, with EHD1 and Rab11-Rab8 forming a complex to mediate this process (Wu et al., 2018). Another study showed that the centrosome protein MICAL like 1 (MICAL-L1) is responsible for recruiting EHD1, removing centriolar coiled-coil protein 110 (CP110) from the mother centrosome, and then promoting cilium formation (Xie et al., 2019). EHD1 knockout mice confirmed that EHD1 positively regulates cilia, which can directly Smo; EHD1 knockout resulted in significant downregulation of GLI3, thereby activating Hedgehog signaling (Bhattacharyya et al., 2016). FGFR3 can mediate intestinal cell kinase (ICK) phosphorylation and inhibit its activity. The activation of fibroblast growth factor (FGF) signaling pathway and the inhibition of ICK activity can jointly regulate the length and function of primary cilia (Kunova Bosakova et al., 2019).

### Age-Related Macular Degeneration

There are two main AMD types, choroidal capillary atrophy and neovascularization, which are closely related RPE cell dysfunction (Pennington and DeAngelis, 2016; Luu and Palczewski, 2018). It has been shown in two cell lines that lack of primary cilia hinders RPE cell differentiation (May-Simera et al., 2018). In addition, BBS8 knockout mice have primary ciliary dysfunction and also show signs of retinal degeneration (Dilan et al., 2018). AMD is characterized by the harmful accumulation of lysosomal lipofuscin in RPE cells, with A2E, the main fluorophore, identified from lipofuscin in the eyes of elderly individuals (Sun et al., 2019). Correspondingly, the growth of RPE cells decreased, the cell morphology changed, and RPE cells became senescent. Cells can only divide when primary cilia depolymerize, and Aurora A expression is necessary for cilium depolymerization. The decrease in caveolin-1 expression degrades Aurora A, preventing primary cilia from disaggregating and inducing premature senescence of human fibroblasts (Jeffries et al., 2019). In AMD, HtrA serine peptidase 1 (HTRA1) expression is upregulated (Lin et al., 2018). In a mouse model of BBS, a ciliopathy, TGF-β1 expression decreased and HTRA1 expression increased (Sheffield et al., 2018). Therefore, lack of primary cilia can lead to dysfunctional RPE cell differentiation, abnormal ciliary depolymerization, and RPE cell senescence.

### Inherited Retinal Diseases

Inherited retinal diseases are progressive, hereditary fundal diseases characterized by damage to retinal photoreceptor cells and RPE cells. The major inherited retinal diseases include non-syndromic retinitis pigmentosa (RP), cone-rod dystrophy, Usher syndrome, and BBS (Takahashi et al., 2018). Due to the ciliated nature of the photoreceptor cells, most known disease-related molecules are closely related to the primary cilia. Rhodopsin is the main component of the outer segment of photoreceptor cells, but rhodopsin is first formed in the inner segment. After formation, rhodopsin forms a complex with Arf4 and Golgi brefeldin A resistant guanine nucleotide exchange factor 1 (GBF1), is transported to the outer segment by connecting cilia, and participates in the phototransduction cascade. GBF1’s selective inhibitor golgicide A can prevent the delivery of rhodopsin to the cilia without damaging the photoreceptor Golgi (Wang et al., 2017).

In addition, peripherin 2 is a main component of the outer segment also formed in the endoplasmic reticulum and Golgi and transported to the outer segment through the cilia (Otsu et al., 2019). RPGR, which is located in the ciliary transition region, serves as a scaffold protein to recruit the small GTPase Rab8A, phosphodiesterase 6D (PDE6D), and Arl3 to carry cargo to the cilia, and PDE6D can also promote the positioning of cilia by prenylating RPGR (Murga-Zamalloa et al., 2010; Dutta and Seo, 2016). It has been shown that the removal of OFD1 by autophagy is a necessary condition to promote the development of mammalian cilia (Tang et al., 2013). Leber congenital amaurosis (LCA) is a typical retinal disease that causes blindness in children. LCA is caused by a mutation in centrosomal protein 290 (CEP290, c.2991 + 1655A > G), which leads to abnormal splicing and inserts the cryptic exon X into the stop codon in the mRNA transcript to inactivate CEP290 protein activity (Parfitt et al., 2016). CEP290 has been widely confirmed to be involved in primary ciliary vesicle transport and transition zone formation (Jana et al., 2018).

## Gene Therapy For Ocular Diseases

Gene therapy describes modifying the normal function of genes or replacing defective genes for therapeutic benefit (Brody, 2018). Cilium-related genes are often lost in recessive eye diseases, so restoring gene expression could improve ciliary function. Gene therapies include gene augmentation, gene replacement, and drug intervention. In autosomal dominant eye diseases, gene silencing technology can be used to suppress mutant genes. Adeno-associated viruses (AAVs) are non-pathogenic vectors that avoid the immune system by penetrating cell membranes (Robert et al., 2017). Therefore, AAV vectors have been widely used as vehicles in gene therapy. The hereditary nature of many eye diseases makes gene therapy an attractive technology. For example, for the treatment of X-linked RP, under the drive of the AAV8 vector and rhodopsin kinase promoter, RPGR^ORF15^ coding sequence optimization has been shown to effectively restore RPGR mutations (Fischer et al., 2017). This approach has now been tested in phase I and II gene therapy clinical trials. Various animal models and clinical trials have also provided evidence that subretinal injection of AAV2-RPE65 restores the impaired rhodopsin regeneration caused by biallelic mutation of the RPE65 gene and restarts the visual cycle (Apte, 2018). However, the traditional AAV vehicle has limited carrying capacity, and DNA fragments of about 4.3 kb cannot be genetically edited using traditional techniques. The double-AAV strategy appears to be able to overcome this limitation; for example, ATP binding cassette subfamily A member 4 (ABCA4) or RP1 genes of more than 5 kb can be transmitted with two different AAV vectors (Auricchio et al., 2015; Nanda et al., 2019).

CRISPR/Cas9 gene editing technology now allows alterations of several thousand base pairs of nucleotide sequence, revolutionizing the field (Araldi et al., 2020). In LCA10 patients, IVS26 mutations of CEP290 have been successfully repaired by CRISPR/Cas9 technology (Ruan et al., 2017). In addition, antisense oligonucleotides can be used to target mRNAs, resulting in their double-strand degradation and restoring correct gene splicing. QR-110 binding to pre-CEP290 mRNA can prevent abnormal splicing, thereby skipping exon X in the mRNA and improving CEP290 wild-type transcript (Dulla et al., 2018). Safety and tolerability studies of intravitreal injection of QR-110 have been completed. Further, artificial zinc-finger protein technology has also been used to transcriptionally silence mutated rhodopsin alleles. Other small molecule drugs have also used been used as gene therapies. For example, trichostatin A, an HDAC inhibitor, not only effectively promotes cilium formation but also inhibits diabetic or TGF-β-induced renal fibrosis, reducing the proliferation of fibroblasts caused by platelet-derived growth factor. This may therefore be useful in the treatment of corneal inflammation (Huang et al., 2019).

## Stem Cell Therapy for Ocular Diseases

Ocular stem cell therapy has received widespread attention (Alio Del Barrio and Alio, 2018). Undifferentiated embryonic stem cells have primary cilia, and IFT88 knock-out reduces the ability of mesenchymal stem cells to differentiate into osteogenic, adipogenic, and chondrogenic cells. Primary cilia have also been shown to be important in the development of embryonic neural progenitors into neural stem cells through the Hedgehog pathway (Tong et al., 2014). Dentate gyrus progenitor cells cannot develop when there are deficiencies in ciliary genes or Smo, and the dentate gyrus expands significantly when SmoM2-YFP is expressed (Han et al., 2008). Interestingly, experiments in mice have shown that the hippocampal subventricular zone of elderly mice retained a pool of neural stem cells, but their proliferative potential was reduced through prolongation of the G1 phase of senescent stem cells, while cell cycling was normal in the remaining active cells. Therefore, the normal formation and resorption of primary cilia play a key role in regulating stem cell proliferation and cellular differentiation, providing a theoretical basis for cilia regulating stem cell differentiation.

In iPSC-RPE, AAV-mediated pre-mRNA processing factor 31 (PRPF31) delivery increased PRPF31 levels several fold without any signs of toxicity, significantly restoring *PRPF31* ^+/−^ mutant cell phagocytosis and cilium length, supporting its potential as a clinical therapy (Brydon et al., 2019). CRISPR/Cas9-mediated *in situ* gene editing technology can also be used to induce normal gene and protein function in stem cells. Mutations in PRPF31 leads to haploid deficiency and incomplete expression, which is a main reason for retinal degeneration (Buskin et al., 2018). This mutation also prevents IFT88 from being transported to the top of the cilia, and coiled-coil and C2 domain containing 2A (CC2D2A) and RPGR-interacting protein 1-like (RPGRIP1L) cannot be recruited into the transition zone. CRISPR/Cas9 modification of mutant PRPF in iPSCs of RP11 patients restored all the key cell and functional phenotypes of the RPE layer, a finding also validated in the RP39 animal model (Foltz et al., 2018). Usher syndrome type 2A (USH2A) mutation was found in iPSCs obtained from patients with Usher syndrome. This mutation could be repaired by the CRISPR/cas9-mediated gene editing, so that photoreceptor progenitor cells differentiated by the iPSCs restore visual functions (Sanjurjo-Soriano et al., 2020).

## Conclusions and Perspectives

There has been an increasing amount of research on primary cilia over recent years, revealing their important biological functions. The functions of ocular cilia, especially with respect to cilium formation and cilium-mediated transport in photoreceptor cells, have gradually become clear. It has been confirmed that primary cilia and related molecules are involved in many eye diseases. Continuing improvements in sequencing and mass spectrometry technologies delivering higher resolution genomics and proteomics through single-cell sequencing and characterization of post-translational modifications are shedding new light on the roles of cilia in eye development and disease. However, several questions remain unanswered. What is the connection between primary cilia and corneal differentiation and retinal development? Why is primary ciliary protein localization in the eye different from other tissues? Are there any new related proteins involved in the development of cilia and eye diseases? How do ciliary function in other parts of the eye? Therefore, further work is needed to establish the relationship between primary cilia and eye diseases as well as determining the importance of the localization and functions of key ciliary proteins in the cornea, retina, and other tissues.

Developments in the speed and resolution of gene sequencing technologies are contributing to the early diagnosis of diseases. Compared with traditional drug therapy, gene therapy can also be used to reverse disease pathogenesis. In particular, CRISPR/Cas9 gene editing technology is now making it possible to fix causative mutations and has also accelerated some single-gene drugs into clinical trials. However, several challenges remain in the clinical application of gene therapy technology, including off-target effects and autoimmune responses. Most eye diseases are not single gene disorders; therefore, sequencing will be required to establish the multiple mutations involved in disease pathogenesis and then gene editing could be used to formulate individualized gene therapy programs. It is likely that several molecular pathways will need to be targeted for disease treatment. Cilia play a vital role in different parts of the eye, and a deeper understanding of the structure and function of cilia is required to develop new treatments for eye diseases.

## Author Contributions

PZ wrote the manuscript and drew the figures. JZ conceived the study and revised the manuscript. All authors contributed to the article and approved the submitted version.

## Funding

This work was supported by the Taishan Scholars Program of Shandong Province (20161201).

## Conflict of Interest

The authors declare that the research was conducted in the absence of any commercial or financial relationships that could be construed as a potential conflict of interest.
